# The promise of refugee lifelong education: A critical review of the field

**DOI:** 10.1007/s11159-021-09927-5

**Published:** 2021-11-09

**Authors:** Linda Morrice

**Affiliations:** Centre for International Education, University of Sussex, Essex House, Falmer, Brighton, UK

**Keywords:** Refugee education, Lifelong learning, Lifelong education, Postcolonial, Refugee

## Abstract

The increasing number of refugees, coupled with the protracted nature of refugee situations around the globe, underline the critical importance of refugee education. Since 2010, education has been one of the global strategic priorities of the United Nations Refugee Agency (UNHCR), but much of the focus and resourcing has been on primary education and, to a lesser extent, secondary education. Recognition of the role of lifelong learning for refugees has been much slower, only recently entering into prominence in global documents and policies. For refugees, the hope and desire for education that will enable them to achieve sustainable and dignified livelihoods has always been a core part of their realities. Lifelong learning has the potential to provide the bridge between disrupted schooling and future aspirations of self-reliance and participation in society. This article situates the emerging agenda for refugee lifelong learning in a postcolonial and global context. The author begins with a critical examination of the postcolonial “logics” which continue to construct and frame the study of refugees, before problematising how lifelong learning is incorporated and conceptualised in global policy. She draws the conclusion that despite global commitments, the realisation of lifelong learning for refugee youth and adults is likely remain frustrated for some time by normative assumptions firmly embedded in the conceptualisation of lifelong learning and the education frameworks of nation states. However, she argues that the recent global disruption to education and the inequalities that have been exposed by the COVID-19 pandemic provide an opportunity to rethink how education is conceptualised and the importance of providing lifelong learning opportunities to enable young people and adults to fulfil their aspirations.

## Résumé

La promesse de l’éducation tout au long de la vie pour les réfugiés : un examen critique de ce secteur – Le nombre croissant de réfugiés ajouté à la durée prolongée de leur situation dans le monde entier souligne l’importance décisive de l’éducation des réfugiés. Depuis 2010, l’éducation est une des priorités stratégiques mondiales de l’Agence des Nations Unies pour les réfugiés (UNHCR). Toutefois l’attention et les ressources ont majoritairement été dévolues à l’éducation primaire et, dans une moindre mesure, à l’éducation secondaire. Reconnaître le rôle de l’apprentissage tout au long de la vie s’est fait beaucoup plus lentement, et ce n’est que récemment que ce secteur a gagné en importance dans les documents et politiques internationaux. L’espoir et le souhait que l’éducation permette aux réfugiés de gagner leur vie durablement et dignement a toujours fait partie intégrante de la réalité qu’ils vivent. L’apprentissage tout au long de la vie pourrait être le pont entre une scolarité perturbée et de futures aspirations à devenir autonomes et à prendre part à la vie de la société. Cet article situe l’agenda naissant de l’apprentissage tout au long de la vie dans un contexte postcolonial et mondial. L’autrice part d’un examen critique de la « logique » postcoloniale qui continue de constituer et de définir l’étude des réfugiés, et pose ensuite le problème de la façon d’intégrer et de conceptualiser l’apprentissage tout au long de la vie dans la politique mondiale. Elle conclut que malgré les engagements pris dans le monde, la réalisation de l’apprentissage tout au long de la vie pour les jeunes et les adultes réfugiés risque d’être encore contrariée pendant un certain temps par les théories normatives fermement ancrées dans la conceptualisation de l’apprentissage tout au long de la vie et les cadres des États-nations en matière d’éducation. Cependant, elle affirme que la récente perturbation mondiale de l’éducation due à la pandémie de COVID-19 et les inégalités que cette dernière a mises en évidence sont pour nous l’occasion de repenser la façon dont l’éducation est conceptualisée et l’importance d’offrir des possibilités d’apprentissage tout au long de la vie aux jeunes et aux adultes pour leur permettre de réaliser ce qu’ils ambitionnent.

## Introduction

The mismatch between the educational aspirations of refugees and the realities of the opportunities available to them is well documented (Dryden-Peterson 2017; Morrice et al. 2019; Shakya et al. 2012). Around the globe, refugees struggle to access education, and the older they become, the greater the challenges. Globally, 63% of refugee children are enrolled in education at primary level, reducing to 24% at secondary level. At tertiary or higher education^1^ level, enrolment is just 3%, compared to 37% of non-refugee students (UNHCR 2019). The education of adult refugees has remained neglected, and their “tertiary education rights often extend to non-discrimination at most” (UNESCO 2018, p. 151). The figures on refugee access to education provide a blunt indication of the dire state of refugee education, but they also hide enormous global disparities. For example, in 2018, global participation in tertiary education ranged from 9% in low-income countries to 75% in high-income countries (UNESCO 2020, p. 239). Most of the world’s refugees (85%) live in low- and middle-income countries where education systems are already struggling to provide access to quality learning opportunities for local children and youth. Very few of the world’s refugees live in wealthier countries in the Global North, and only a tiny number (less than 1%) will enter the North through refugee resettlement schemes (UNHCR 2020).

This article maps the emergence of refugee education and, more recently, lifelong learning,^2^ in humanitarian and development responses. While growing recognition of the importance of lifelong learning for refugees is welcome, I suggest that the intersection of the study of refugees and education requires a critical framing, with both “refugee” and “lifelong learning” opened up to question and interrogation.

A significant body of academic literature examines the lack of educational opportunity structures for refugee adults and young people in countries in the Global North (e.g. Earnest et al. 2010; Kanno and Varghese 2010; Morrice 2013; Stevenson and Willott 2007), and a much smaller, but growing, literature investigates this lack in the Global South (e.g. Avery and Said 2017; Bellino and Dryden-Peterson 2019; Crea and McFarland 2015; Dryden-Peterson 2010; Kamyab 2017). With a few notable exceptions (Dryden-Peterson, 2010, 2016; Ramsay and Baker 2019), the vast majority of literature focuses on national contexts. Most is also only concerned with *either* the Global South *or* the Global North. The focus is often on what refugees as newcomers lack in order to progress in that national context, and the literature highlights barriers that include required documentation, host community language, qualifications which are recognised and required to progress, academic and literacy skills, and curricular knowledge. Additional barriers in the Global South include poor infrastructure, such as a low number of colleges and the distance to those colleges, and reliability of power supply (UNHCR 2019). Less critical attention has been paid to the structural barriers created by national education systems into which refugees become inserted (Morrice et al. 2019). As lifelong education for refugees gains recognition, it is timely to examine how it is conceptualised, the purpose it serves and whether it can fulfil that purpose.

The field of refugee studies has been criticised for an ahistorical, apolitical framing which has led to a lack of critical questioning (Chimni 1998; Malkki 1995; Mayblin 2017). Concepts and categories such as “refugee” and “nation state” have assumed a taken-for-granted naturalness which can render invisible the purposes such categories serve and their consequences for the lived experiences of refugees and other non-citizens. While understanding national context and the complex relationship between states and education systems is important, taking the nation state as the only or primary unit of analysis blinds us to the overarching commonalities in the challenges facing refugee education in both the Global North and South.

I argue that although middle- and low-income countries face far greater challenges in fulfilling the international commitment to quality education for all, many of the struggles that refugees face result from the same structural barriers and systematic exclusions in national education systems wherever they are located. To reveal the abiding regime of inequality and lack of opportunity structures, I adopt an approach which transcends traditional North–South division and the nation state as dominant categories. It is an approach which aligns with the universal frame of reference of the United Nations Sustainable Development Goals (SDGs), which mark a radical shift from the Millennium Development Goals’ exclusive focus on “developing” countries in the South (Horner 2020). This approach also reflects growing inequalities within many countries and patterns of “converging divergence”, where there is some convergence between countries of the Global North and South, alongside divergence within countries (Horner and Hulme 2019). Postcolonial theory has long called into question North–South (or Western–non-Western) binaries (e.g. Said 1979; McEwan 2009; Santos 2016). Cheryl McEwan (2009), for example, calls such distinctions “metaphorical” rather than geographical, and Boaventura de Sousa Santos (2016, p. 127) refers to a “messy cartography”, highlighting how the systematic exclusion and suffering of marginalised populations can be found in both North and South, and within wealthy nations. Refugees form one such marginalised population.

The distinction between refugees in the Global North and South is also becoming increasingly blurred (Dryden-Peterson 2016). Large numbers of refugees entered Europe in 2015/2016, and many have found themselves caught in limbo in refugee camps or urban areas in southern Europe, particularly Greece and Turkey. Tens of thousands of asylum seekers^3^ also arrive every year in Europe; they face similar uncertainty about their right to remain and similar restrictions in their right to education and employment as refugees in host countries in the Global South. Even refugees who arrive in the Global North through resettlement schemes with pathways to citizenship face educational challenges which continue long after their arrival (e.g. Morrice et al. 2019; Nunn et al. 2017). These refugees and asylum seekers face exclusions and barriers similar to refugees and asylum seekers in countries in the South, yet in the context of the resource-rich countries of the North.

As a result, this article aims to:

1. challenge the taken-for-granted assumptions and framing implicit in discussions of refugees and education, in order to lay the groundwork for more critical approaches; and
2. probe the conceptualisation and assumed purpose of lifelong education for refugees as it emerges as a contemporary priority in documents produced by the United Nations Refugee Agency (UNHCR).

Meeting the challenge of enabling lifelong learning for refugees requires a re-think of lifelong education conceptualised as an opportunity located at the end of a set of established transitions between educational levels and occurring at designated points in the life course. Rather, it requires positioning refugees’ goals and aspirations for dignified and self-sufficient lives as the starting point, and then considering what structures and support are required to enable these goals to be fulfilled.

The discussion in this article begins by locating refugee studies within the colonial legacies and postcolonial “logics” of the European nation state. I draw on the work of postcolonial scholars to critique both the nation state and the concept of “refugees” as unproblematic categories. The refugee is situated historically in the emergence of the United Nations *Convention relating to the Status of Refugees* (UN 1951; henceforth referred to as the “Refugee Convention” or simply “Convention”)^4^ from a post-Second World War colonial/postcolonial viewpoint. This approach provides a global perspective which reveals the commonalities in refugee education across nation states and North–South binaries. It also enables us to understand the “contemporary tension between the global promise of these rights [to education] and their limited realisation within nation-states” (Dryden-Peterson 2016, p. 475). Having established this critical understanding, in the subsequent sections I trace the emergence of education and, more recently, opportunities for lifelong learning as a global priority for refugees. Conceptualisations of lifelong education as the final stage in an educational journey from primary to secondary to tertiary level reflect the normative assumptions and “sedentarist”^5^ principles (Malkki 1995) of national education systems, which I suggest represent a missed opportunity to provide meaningful opportunities for refugees.

## Postcolonial construction of the nation state and the refugee

### The nation state

The nation state is foundational to the *Refugee Convention’s* definition of a refugee as a person who “is outside the country of his [*sic*] nationality” (UN 1951, Article 1 A(2), cited in UNHCR 2010, p. 14). The nation state is also key to determining the rights and opportunities available to refugees, including the right to education. The assumption that the nation or state is the natural social and political form of the modern world has been central to theory and methodology in mainstream social science for more than a century (Wimmer and Glick Schiller 2002). Such state-centric framing imagines citizens/non-citizens as congruent with territorial national borders and reproduces “the starting premise of white nationalism: namely, that migrants are ‘strangers’, ‘charitable subjects’, and ‘uninvited guests’” (Danewid 2017, p. 1675).

Postcolonial scholars have challenged the uncritical adoption of these national frames, not only because they are based on European models of the nation state, but because they also ignore the role of empire and colonialisms in the formation of the contemporary world. A substantial body of literature has shown how national borders are constructed, maintained and correlate to the logics and legacies of empire (Anderson et al. 2009; Davies and Isakjee 2018; De Genova 2010; Walia 2013). Gurminder Bhambra (2016), for example, demonstrates how the European nation state is a colonial and imperial state whose dominance was based on extraterritorial extraction, dispossession and enslavement. Yet, she argues, how colonial relations connected the formation of European nation states with their colonial and imperial territories overseas is rarely theorised as a constitutive aspect of the state. Neither is the way in which the refugee as non-citizen is historically produced, often through the dismantling of European colonialism. A number of scholars have outlined how the large-scale displacement of people is not coincidental, but rather created through a long history of empire, colonial conquest and slavery (Danewid 2017, p. 1680; Gregory 2004; Mayblin 2017; Walia 2013).

### The refugee

European dominance, colonial histories and logics of the mid-twentieth century are deeply rooted in the current refugee regime, the definition of a refugee and its exclusions (Chimni 1998; Malkki 1995; Mayblin 2017). European states and their vision of the world were the driving force behind the *Refugee Convention* in 1951 and its 1967 *Protocol* (UN 1951, 1967). Emerging from large-scale displacement following the Second World War, the *Refugee Convention* expressed Eurocentric concerns over growing communism, preventing another holocaust and individual persecution (Hyndman 2000). These European concerns are reflected in the *Convention*’s definition of a refugee as a person who

owing to well-founded fear of being persecuted for reasons of race, religion, nationality, membership of a particular social group, or political opinion, is outside the country of his [*sic*] nationality (Article 1 A(2), cited in UNHCR 2010).

This definition is meanwhile recognised as being outdated and increasingly problematic for the demands of the contemporary world, and particularly for populations in the Global South (Betts^2013^; Betts and Collier 2017; Hyndman 2000; Zetter 2015). For example, this definition establishes “persecution” as an archetypal driver of forced migration; yet, a great many people are forced into exile for reasons which cannot be reduced to individualised or even group persecution (Betts 2013; Zetter 2019). Many other complex and overlapping concerns and drivers of forced migration are also excluded from this definition. R﻿﻿oger﻿﻿ Zetter﻿ (2019) provides a typology of five broad scenarios of forced migration: (1) economic precarity of life in fragile states; (2) armed conflict; (3) development-induced displacement (e.g. urban renewal projects and dam-building); (4) natural disasters; and (5) environmental degradation and climate change-related movements. As individual persecution is not the main driver of this migration, forcibly displaced people from these scenarios (most of whom originate in the Global South) fall outside the established international legal and normative frameworks established by European states, and are often categorised as “voluntary” and/or “economic” migrants (Betts 2013; Crawley et al. 2018). The fact that many refugees originate from poor countries, often ex-colonies, inevitably leads to blurred boundaries between so-called economic and persecution drivers, further amplifying the difficulties that people from non-Western and non-settler nations face in claiming protection and accessing rights (Mayblin 2017).

Lucy Mayblin demonstrates how although the *Refugee Convention* is often presented as “transformative”, it provided the first legal framework for colonial relations. The Convention’s definition cited above was based on the assumption that most refugees in the world were European, despite the mass displacements occurring in other parts of the world as a result of the dismantling of colonialism. For example, the mass displacement of an estimated 10 million people in the wake of the partition of India in 1947 was not recognised as being of immediate concern to the Convention. Indeed, Mayblin sets out in detail how Britain and other colonial states ensured that the Convention excluded the colonies, arguing that those outside Europe required “different solutions”. These solutions provided financial aid to ease some of the burden on host countries, but did not recognise displaced people in other parts of the world as refugees. Furthermore, colonialism and associated practices were not considered as persecution; people living in colonial territories could not claim asylum from the injustices of colonial rule, since these human rights were reserved only for non-colonised people. Thus, while the Convention is often presented as transformative and as representing the cementing of universal human and refugee rights, in effect, it established a practical regime of exclusion, differential policy treatment and unequal access to rights (Mayblin 2017). It was not until 16 years later that the application of the Convention was eventually extended with a new protocol (UN 1967) which expanded its scope to all refugees, not just those fleeing Europe (Malkki 1995).

However, the pervasive “myth of difference” persists which perpetuates the idea that there is a fundamental difference between the European refugee movements of the past and those of the present (Chimni 1998). This myth enabled the construction of the “normal refugee … – white, male and anti-communist – which clashed sharply with individuals fleeing the third world” (Chimni 1998, p. 351). Privileging the European subject is not based on the legitimacy of an individual’s claim, but rather on his or her country of origin. The myth has given rise to strategies for containing refugees in the Global South, and it continues to be used to justify differential access to rights for migrants (Chimni 1998, 2009; Mayblin 2017). It is this coloniality which “explains the impasse between the theoretical rights-bearing human and the lived reality of the ‘other’ who struggles to access the right to asylum” (Mayblin 2017, p. 39). Not only does the current refugee regime contain deeply embedded inequalities which leaves large populations with no international protection or access to rights, but, as Ida Danewid (2017) argues, the Convention also sustains the vision of the West as a bastion of democracy and universal rights, and perpetuates the “patronising fantasy of the white man’s burden – based on the desire to protect and offer political resistance *for* endangered others” (ibid, p. 1675, emphasis in the original).

The legal definition of a refugee has always been partial and designed to serve the interests of national state policy (Chimni 2009). A significant body of migration literature has critiqued the legal category and its exclusions (see Koser and Martin 2011). Most notably, Zetter (1991) has demonstrated how the proliferation of legal categories (such as “asylum seeker” and “humanitarian protection”) have resulted in differential policies towards those who do, and those who do not, qualify for the label. Heaven Crawley and Dimitris Skleparis (2018) highlight how politics and self-interest lie at the heart of how states interpret and apply the Convention’s definition, arguing that choosing to label someone “as a ‘refugee’ is a powerful, and deeply political process” (Crawley and Skleparis (2018, p. 52). Richard Black warns against uncritically accepting the category of refugee, arguing that

at best, the term simply reflects the designation of refugee enshrined in a particular Convention at a particular time, within a particular international political and economic context (Black 2001, p. 63).

He goes on to warn of the “perception of the naturalness of the category” which the term invokes and the differential policies towards those who do not qualify for the label (ibid.). The term “refugee”, therefore, does not denote an “objectively self-delimited field of study” and there is no “single essential ahistorical refugee condition”, merely a mixed category of people who have been ascribed the same legal status (Malkki 1995, p. 496). A substantial body of literature demonstrates the complexity of forced migrant lives, and how they do not fit neatly into conceptual and policy categories (Crawley and Skleparis 2018). As others have pointed out (Collyer and de Hass 2012; Crawley and Skleparis 2018), categories and labels are inevitable, and shifting the boundaries simply creates new ones. Thus, it is not my intention to suggest that we do not use the term “refugee”; rather, it is important that we foreground a critical awareness of the constructedness of the category and the consequences for the lived experiences of displaced people.

This section has demonstrated how the international refugee regime is inseparably bound to the European colonial project. It has highlighted how access to fundamental rights (such as rights to asylum), and therefore to rights which flow from this (such as rights to education), is not universal, but is constructed in deeply political contexts which take for granted the nation state as the unit of analysis. The implications of this analysis for refugee education are threefold.

First, as Liisa Malkki eloquently argues, territorialising concepts of nation states produces a powerful sedentarism in our thinking – “a sedentarism that is taken for granted to such an extent that it is nearly invisible” (Malkki 1992, p. 31). This sedentarism not only frames refugees as “pathological” and not “ordinary people” (ibid., p. 33), but also allows the conceptualisation of education systems based on sedentarist principles to flourish unchallenged. National education systems are based on normative assumptions that children progress relatively smoothly through a system and achieve national certification at key points, which enables them to progress to tertiary or higher education. Transnational migrancy in any form challenges this sedentarist framing, but in the case of refugees where arrival is unplanned and unpredictable – and where there is a likelihood of missed education, language barriers, no certification or certification which is not recognised – the challenge to the system is acute. Even in resource-rich education systems such as the United States (US) and United Kingdom (UK), research indicates that young refugee students struggle to catch up in time to acquire the certification to progress to post-compulsory education with their peers or before becoming “over-age” (Bonet 2018; Morrice et al. 2019). In low-resource contexts, the struggle is that much greater.

Second, it is important to ensure that our thinking and research are not constrained by constructed categories such as “refugee”, and these categories should not be allowed to shape our understanding of the social world (Malkki 1995). Rather, we need to challenge the boundaries between categories and differential rights assigned, and to resist notions that some people are more “deserving” than others (Crawley and Skleparis 2018). In both research and practice, this requires attention to who is included/excluded from research agendas, who has the “right paperwork” and is in the “right category” to access lifelong learning opportunities, and who can and cannot use their education to create sustainable livelihoods.

Third, and related to this, there is a need to recognise the disjuncture between the lived reality and needs of displaced people and the legal and policy categories. Educational aspirations and needs vary enormously and cut across administrative categories. Personal histories, socioeconomic backgrounds and contexts are among the many factors which lead to qualitatively different experiences and needs. Accepting that the term “refugee” is not analytically useful for denoting a particular kind of person who is qualitatively different from other forced migrants suggests the need to focus on lived experience (Crawley and Skleparis 2018; Malkki 1995) and to push for a “needs-based” (Zetter, 2019) and inclusive approach to refugee education. A grounded, contextual approach which starts with refugees and their aspirations and needs must be at the centre of the design and delivery of educational programmes.

Having established this critical framing, in the next section I briefly outline the emergence of education as a priority in humanitarian contexts and, more recently, the recognition of lifelong learning in international instruments and policy discourses, before returning to this discussion and offering a number of concluding thoughts.

## The emergence of refugee education

The rise of refugee education is connected to international instruments and embedded in shifting understandings of the purposes of education (Dryden-Peterson 2011). In the context of refugees, education is under the mandate of and steered by UNHCR,^6^ a multilateral institution, but the mechanisms of enforcement are administered by the nation state (the host country) and are “deeply dependent on the relationship between the population to be educated and the nation-state” (Dryden-Peterson 2016, p. 476). Early articulations of refugee education focused on providing basic or primary education, with other forms of education (secondary, vocational, technical or higher education) either not mentioned at all or given a much lower priority in international documents. This imbalance is evident, for example, in Article 22 of the 1951 *Refugee Convention*, which notes that signatory states

shall accord to refugees the same treatment as is accorded to nationals with respect to elementary education … [and] shall accord to refugees treatment as favourable as possible, and, in any event, not less favourable than that accorded to aliens generally in the same circumstances, with respect to education other than elementary education (cited in UNHCR 2010, p. 24).

This focus on basic education is also reflected in other international instruments. For example, it is set out in the *Universal Declaration of Human Rights* (UN 1948), which stipulates the right to free and universal education at the elementary stage, but states that

[t]echnical and professional education shall be made generally available and higher education shall be equally accessible to all on the basis of merit (ibid., Article 26.1).

Despite recognising the right to education, historically very few resources within UNHCR have been allocated to education. It was only from the late 1980s, and in response to increasing numbers of refugees, that refugee education gained traction (Dryden-Peterson 2011). The 1990s saw numerous conflicts around the globe and the role of education both during emergencies and in early reconstruction became an increasingly important concern for the international community (Kagawa 2005). In 1989, the UN *Convention on the Rights of the Child* (UNCRC; UN 1989) set out access to education as an inalienable right of all children and was adopted as a normative framework by UN agencies. In 1995, UNHCR issued *Revised Guidelines for Educational Assistance to Refugees* (replacing an earlier version issued in 1992), affirming its commitment to ensuring that the “ladder of educational opportunity” is made “available and accessible” to every child (UNHCR 1995, p. iv).

A much weaker commitment to access to higher education was made “on the basis of capacity by every appropriate means” (ibid., p. iv). The UNHCR *Guidelines* at the time made clear that attending university or similar post-school training would be open to only a very small number of students and subject to available funds. Students who completed secondary education could apply for a small number of scholarships under the DAFI programme,^7^ and vocational education was to be targeted to vulnerable groups, such as people with disabilities and female-headed households (ibid., p. iv). The *Guidelines* accept the inevitability of “an educational pyramid” (ibid., p. 5) with a broad base (the lower years) and a narrow top (secondary education and beyond), with more boys than girls participating at every level. Despite the inevitable dislocation of education trajectories in displacement contexts, the guidelines do not include any provision for young people who may have had their education interrupted and not been able to complete secondary education, or for adults. Higher levels of education were either overlooked or resisted in a global education movement which prioritised primary and secondary education (Dryden-Peterson 2010).

In the same decade, the influential “Machel report” on the *Impact of Armed Conflict on Children* (Machel 1996), was submitted to the United Nations (UN). The report called for education to be established as “a priority component of all humanitarian assistance”, including reconstruction activities, and for donors to extend the boundaries of emergency funding to include support for education (Machel 1996: para 190, p. 55). Graça Machel’s report was endorsed by the UN which recommended that nation states, UN agencies and non-governmental organisations (NGOs) should provide children with educational opportunities at both primary and secondary levels (Sinclair 2001).

The Machel report is generally recognised as embedding education as the fourth strand of humanitarian responses, alongside food and water, health care and shelter (Kagawa 2005) – although it was the protective function of education that tended to be emphasised, and education was still only understood as a priority at primary and secondary levels. In line with what was at the time the favoured durable solution to refugee situations, it was assumed that education was geared towards voluntary repatriation to a refugee’s country of origin (UNHCR 1995). Refugee education was therefore generally delivered in parallel provision to national systems and was often based on the curriculum of the country of origin and frequently uncertified. Lifelong learning was not considered, as it was implicitly assumed that refugees would return to their home country and resume their adult lives there.

## Out of the shadows: lifelong learning

Increases in the scale and protracted nature of refugee situations in the 2000s, and recognition that most refugees will not quickly return to their country of origin (UNHCR 2019), led to changes in the conceptualisation of refugee education and what purpose it should serve. Education could no longer be assumed to be a temporary “stopgap” measure; instead, it required a longer-term perspective which imagined refugee students as eventual contributors to their family or local economy. As a result of these displacement realities, education not only increased in importance, becoming one of UNHCR’s global strategic priorities and a “core component” of its mandate (UNHCR 2012, p. 7), but crucially, the need for lifelong learning opportunities which could prepare refugees for future participation in their host society began to be recognised. Both education throughout the lifecycle and higher education were at last recognised as a “critical part of the educational continuum” (UNHCR 2012, p. 22).

The UN 2030 Agenda for Sustainable Development was launched in 2016, pledging to ensure that no one was left behind (UN 2015). The Agenda’s fourth goal (SDG 4) commits to “… promot[ing] lifelong learning opportunities for all”, and identifies three targets for technical and vocational education and training (TVET) (targets 4.3, 4.4 and 4.5),^8^ and one for improving literacy and numeracy for youth and adults (target 4.6).^9^ To achieve these global goals, the challenge of providing lifelong learning opportunities for the rapidly increasing numbers of refugees needs to be addressed.

Aligning with SDG 4, the framework of the *Global Compact on Refugees* (GCR) (UN 2018) affirmed the importance of providing lifelong learning opportunities, including tertiary education, as a critical element of the international refugee response. Although not legally binding, the *Global Compact* established the international community’s ambition to strengthen solidarity with refugees and host communities in the Global South through more equitable responsibility and burden-sharing. It calls on states and relevant stakeholders to commit resources to expanding and strengthening the quality and inclusiveness of national education systems for both host and refugee students, including TVET. The *Global Compact* also calls for support in recognising the equivalency of academic, professional and vocational qualifications (UN 2018).

The principles and arrangements set out in the *Global Compact* are translated into actions in UNHCR’s updated refugee education strategy, *Refugee Education 2030: A Strategy for Refugee Inclusion* (UNHCR 2019). It establishes three strategic objectives for refugee education: the inclusion of refugee children and youth in the national education system of the host community; the support of learning for all students regardless of legal status, gender or disability; and enabling all learners to use their education towards sustainable futures. The strategy sets out the target of increasing tertiary education enrolment from the current 3% of college-eligible refugees to 15% in host and third countries^10^ by 2030. The primary barrier to tertiary education enrolment is cited as “the limited number of eligible refugee secondary school graduates” (UNHCR 2019, p. 13).

This focus on tertiary education and, in particular, the setting of modest targets, is welcome. However, the emphasis on college-eligible secondary school graduates is deeply problematic in the context of refugee education. Tertiary education comes at the end of well-documented cumulative education disadvantage that prevents many from having acquired the necessary qualifications when they reach school-leaving age. At the point of entry to tertiary education, the structural barriers are manifold, including documentation and credentials required, cost of courses, achievement of the required standard of the host country language (if applicable), and more often than not the need to earn a living. Degrees of acuteness may vary, but the nature of the barriers is the same across the Global North and South, and across national education systems.

Conceptualising lifelong learning as part of a continuum for those who have accessed and progressed through educational levels provides an important incentive for students to continue and complete secondary education; however, at the same time it also effectively closes down the pipeline to tertiary education for most refugees. The concept of entry into tertiary education via alternative pathways, or lifelong learning which prepares refugee youth to become “college-eligible”, does not appear as a priority. Apart from basic literacy and numeracy, tertiary education in global education agendas is not about providing the opportunity structures and support for youth and adults to catch up. Furthermore, while setting targets is an important and welcome step, there is an invisibility of data on refugees when it comes to monitoring progress in achieving the SDGs. There is what Allison Grossman and Lauren Post (2019) refer to as an “SDG refugee gap”, where data on refugees are virtually absent from monitoring frameworks and national reporting. For example, only data on primary education enrolment are consistently available, while data on actual attendance and learning outcomes are very limited. Consequently, there are few mechanisms or systems in place to ensure that global commitments enshrined in the SDGs and the *Global Compact* are translated into practice at the national level.

## Discussion and conclusion

In response to protracted displacement, the purpose of refugee education has shifted from an initial focus on providing “normalcy” and protection for children (INEE 2004), to more recent recognition of the need to prepare refugees for “participation in cohesive societies” (UNHCR 2019, p. 6). Opportunities for tertiary education are now seen as critical to refugees. The prospect of being able to join higher education not only provides an incentive for children and young people to remain in school, contributing to school enrolment and retention (UNHCR 2012); it is also crucial for providing refugees with the means to become self-reliant and to build dignified and sustainable futures for themselves and their families.

These developments, alongside the shift to including refugees within national education systems, are welcome. However, national education systems are based on predictability, linearity and continuity. They are designed as a continuum and assume a sedentary national population moving smoothly through various stages until they graduate from school and transition to the next stage. Lifelong learning for refugees, however, can only fulfil its potential if the fractured educational trajectory of refugee students is acknowledged at the outset, and if the opportunity and support structures for refugees are built into the system. This involves consideration of what skills and knowledge refugee youth and adults require to navigate and build sustainable futures for themselves and their families.

Writing about school education, Sarah Dryden-Peterson (2019) argues for a future-oriented approach to refugee education, which begins with the imagined futures of refugees and then plans backwards. It is an approach which focuses on aspirations and how the system might accommodate the needs of refugee students, rather than students being expected to accommodate the system. In terms of lifelong learning, this involves starting with what refugees need if they are to play an active part in society, including their living dignified and self-reliant lives, and then working back to consider how lifelong education can be reconceptualised in order to fulfil this need.

I have argued for a critical approach to refugee lifelong education which recognises the ways in which the colonial past is still active in the inequalities of the present (Mignolo 2011). From the outset, we need to be clear that the concept of a refugee, and therefore the field of refugee education, is not neutral. To accept a depoliticised and ahistorical account of refugees and their rights to education is to become implicated in the exclusions and colonial ordering of migrants. The colonial present is constitutive of education systems and a critical part of analysis. I have also argued that to only focus on the nation state creates too narrow a lens through which to understand refugee education; it limits our imagining and masks some of the global similarities in the challenges to refugee lifelong education. While taking a global perspective does not entail smoothing out or ignoring the very real differences or experiences in different contexts, it does enable us to see the struggles of refugees around the globe in a more unified way. It illuminates global patterns and gives greater analytical visibility to the problems of achieving global promises for refugee education, placing attention on national education systems and their inflexibilities and failures.

There are vast differences in the scale of the challenges faced by often impoverished countries in the geographical South, whose infrastructures are under enormous strain with the influx of refugees from neighbouring countries. However, we can identify a number of empirical themes: (1) inflexible national structures which do not address learning gaps caused by interruptions to education; (2) lack of social and emotional support; (3) non-recognition and transferability of documentation and qualifications; and (4) lack of language and cultural support. Around the globe, refugees are expected to fit into education systems and structures based on assumptions of an uninterrupted learning continuum. The tendency with this approach is that “the problem” becomes the refugees, their differences and what they lack, rather than the nation state, the structure of education systems, and what these lack. The importance of lifelong learning is amplified for refugees because of its potential to support them in their efforts to catch up with education and opportunities missed at an earlier stage, providing a bridge to self-reliance. It has a key role in enabling sustainable and dignified lives, but this requires a pivot from notions of being “college-eligible” and having the “right qualifications”, to focusing on how lifelong learning can prepare students for the future they aspire to, and working backwards from that point.

At the time of writing, we are in the grip of a global pandemic, and it is interesting to note the emergence of agendas for addressing learning loss and the social and emotional needs of children who have been out of school for many months. The phenomenon of COVID-19 has interrupted education for children, young people and adults around the globe and exposed national education systems in the North to challenges which are common for refugees. In doing so, the pandemic has revealed many of the same inequalities which impact education in the South – the gulf between rich and poor, between those who have and those who do not have access to support, infrastructure and the internet, and how these inequalities intersect with privilege, gender, race and disability.

The disruption to the status quo for non-migrants has raised the question of how to support the most disadvantaged and how to address the significant interruptions to learning. The UK, for example, has launched a national programme to help school students catch up,^11^ and many assessments for 16- and 18-year-olds have either been replaced or scrapped. Universities and tertiary colleges have lowered their entry requirements (Busby 2020), and there is ongoing discussion about how to address gaps in learning. These are stopgap measures in response to a pandemic, but they can also provide an opportunity to rethink how education is conceptualised, the purposes it serves and, in particular, the role that lifelong learning can play in enabling all young people and adults to fulfil their aspirations.

## Footnotes

1 The terms “tertiary” and “higher education” are used interchangeably by the United Nations Refugee Agency (UNHCR) to refer to all post-secondary education, including university degrees and technical and vocational education and training (TVET).

2 While there is no universally agreed definition for the term *lifelong learning,* one of its main features is learners’ active engagement not only in formal but also in non-formal and informal learning throughout their lives. In this article, I use the somewhat less common term *lifelong education* to emphasise the importance of the *provision* of intentional and supported tertiary education programmes and opportunities for refugees. See Jarvis (2004) for a discussion of the emergence of these concepts.

3 An *asylum seeker* is a person who has applied for protection as a refugee in their host country and is awaiting the determination of their status.

4 “The United Nations Convention relating to the Status of Refugees, adopted in 1951, is the centrepiece of international refugee protection today. The Convention entered into force on 22 April 1954, and it has been subject to only one amendment in the form of a 1967 Protocol, which removed the geographic and temporal limits of the 1951 Convention”(UNHCR 2010, p. 2).

5 “Sedentarism treats as normal stability, meaning and place, and treats as abnormal distance, change, and placelessness” (Sheller and Urry 2006, p. 208). The concept (Malkki 1992) is discussed later in this article.

6 Originally, the United Nations Educational, Scientific and Cultural Organization (UNESCO) held the mandate for refugee education. Responsibility was formally transferred to UNHCR in 1967 (see Dryden-Peterson 2011). The United Nations Relief and Works Agency for Palestine Refugees in the Near East (UNRWA) holds the mandate for protection and provision of services for more than 5 million Palestinian refugees. The work of UNWRA is beyond the scope of this article.

7 “The DAFI [Deutsche Akademische Flüchtlingsinitiative Albert Einstein] (Albert Einstein German Academic Refugee Initiative) scholarship programme offers refugee students the possibility to pursue an undergraduate degree in their country of asylum” (UNHCR 2017, p. 2). This initiative is funded by the Federal Republic of Germany.

8 By 2030, SDG target 4.3 aims to “ensure equal access for all women and men to affordable and quality technical, vocational and tertiary education, including university”; target 4.4 aims to “substantially increase the number of youth and adults who have relevant skills, including technical and vocational skills, for employment, decent jobs and entrepreneurship”; and target 4.5. aims to “eliminate gender disparities in education and ensure equal access to all levels of education and vocational training for the vulnerable, including persons with disabilities, indigenous peoples and children in vulnerable situations” (UN 2015).

9 By 2030, SDG target 4.6 aims to “ensure that all youth and a substantial proportion of adults, both men and women, achieve literacy and numeracy” (UN 2015).

10 Refugee resettlement to third countries is one of three durable solutions identified by UNHCR. Refugees resettled to a “third country” (i.e. neither their own country nor the country they originally fled to, seeking asylum) have the right to reside there for a long-term period or even permanently.

11 The programme is called the “National tutoring programme” (NTP). For more information, visit https://www.gov.uk/government/publications/national-tutoring-programme-ntp/national-tutoring-programme-ntp [accessed 22 October 2021].
